# Food and Nutrition Literacy: Exploring the Divide between Research and Practice

**DOI:** 10.3390/foods12142751

**Published:** 2023-07-19

**Authors:** Paula Silva

**Affiliations:** 1 Laboratory of Histology and Embryology, Department of Microscopy, School of Medicine and Biomedical Sciences (ICBAS), University of Porto (U.Porto), Rua Jorge Viterbo Ferreira 228, 4050-313 Porto, Portugal; psilva@icbas.up.pt; 2 iNOVA Media Lab, ICNOVA-NOVA Institute of Communication, NOVA School of Social Sciences and Humanities, Universidade NOVA de Lisboa, 1069-061 Lisbon, Portugal

**Keywords:** health literacy, nutrition literacy, food literacy, nutrition, research patterns and tends, VOSviewer, Harzing’s Publish or Perish

## Abstract

This study addresses the growing recognition of the importance of food and nutrition literacy, while highlighting the limited research in this field, particularly the gap between research and practice. A bibliometric analysis of publications on food and nutrition literacy research from the Scopus database was carried out. Endnote 20, VOSviewer, and Harzing’s Publish or Perish were used to analyze the results. The growth of publications, authorship patterns, collaboration, prolific authors, country contributions, preferred journals, and top-cited articles were the bibliometric indicators used. Subsequently, articles aimed at measuring food or nutrition literacy-implemented programs were analyzed. Existing studies have primarily concentrated on defining and measuring food or nutrition literacy. Although interventions targeting food and nutritional literacy have shown promise in promoting healthy eating, further research is required to identify effective approaches in diverse populations and settings. This study emphasizes the need for additional research to measure intervention program efficacy to enhance the policies and practices in this critical area of public health. These findings underscore the importance of understanding food/nutrition literacy and developing effective interventions to promote healthy eating habits. By bridging the research–practice divide, this study provides valuable insights for policymakers, practitioners, and researchers to address the gaps and improve food/nutrition literacy in various contexts.

## 1. Introduction

Food and nutrition literacy are critical concepts that have acquired increasing attention in recent years. Food literacy comprises a variety of connected knowledge, skills, and actions to determine, manage, pick up, prepare, and consume food. It is having the capacity to make decisions that improve one’s health and contribute to a sustainable food system while considering all social, environmental, cultural, economic, and political variables [1,2,3,4,5]. Nutrition literacy is the degree to which a person can obtain, process, and grasp basic dietetic information and services to make healthy food choices. It entails understanding nutritional concepts and having the capacity to comprehend, evaluate, and apply nutrition information, i.e., to be aware of the nutrients and their impact on health. It concerns an individual’s ability to gather, comprehend, and apply dietary data from various sources. Nutrition literacy also involves knowing how foods are metabolized, how they affect health, and how to use this knowledge to make good decisions [5,6,7,8,9]. According to Krause et al. (2016), food and nutrition literacy are distinct types of health literacy [5]. The authors claimed that nutrition literacy is the competence to understand fundamental nutritional knowledge, which is a prerequisite for a range of abilities classified as food literacy. The authors recommended the use of food literacy rather than nutrition literacy since it is more inclusive and comprises the knowledge and abilities required for healthy and responsible eating [5].

The importance of food and nutrition literacy lies in its potential to promote healthy eating habits and prevent chronic diseases such as obesity, diabetes, and cardiovascular disease. Enhancing individuals’ ability to make informed decisions about their food choices can help reduce the burden of these diseases and improve overall health outcomes [10]. Despite the growing recognition of the importance of food and nutrition literacy, there is limited research on this topic. Most studies have focused on measuring individuals’ levels of food and nutrition literacy and identifying the factors that influence these levels. However, ongoing discussions on definitions and measurement methods can result in delays in the implementation of interventions. This lack of consensus on the best approach to define and measure food and nutrition literacy has hindered progress in the field and created missed opportunities for intervention implementation. Moreover, while there is some evidence that food and nutrition literacy interventions can be effective in promoting healthy eating habits, more research is needed to identify the most effective approaches for different populations and settings. Overall, there is a need for more research on food and nutrition literacy to improve policies and practices in this critical area of public health.

Food and nutrition literacy has become an increasingly important research area in recent years. Many studies have been published on this topic, covering a range of issues, such as nutrition education, food labeling, and dietary behavior change. It is crucial to evaluate the underlying reasons for this wave in the number of research papers on food and nutrition literacy. By doing so, we can ensure that researchers’ efforts to promote food and nutrition literacy are based on sound scientific evidence and are not merely driven by a trend or the publish-and-perish effect. The evaluation of the reasons beyond the wave in research papers on food and nutrition literacy would not only help us gain a better understanding of the current state of research in this field, but also provide valuable insights into the underlying factors that drive this tendency.

In this study, the Scopus database was used as the first approach to conducting a bibliometric analysis of food and nutrition literacy research. It is crucial to note that the Scopus findings are only a small portion of the overall production on this subject, and that the scientific literature is likely to be much larger. In addition, new research is being published daily. Publication growth over time was analyzed to identify trends and study outlines, the authorship pattern to understand collaboration and communication practices among researchers, and the country’s contribution to spotting the geographic distribution of research activity. Moreover, prolific authors were identified to highlight the most active contributors to the field, the most active countries to highlight the ones that are leading the research in the field, and the preferred journals to realize the dissemination and impact of research output. Finally, the top-cited articles were analyzed to discover the most influential research in the field. In food and nutrition literacy research, it is essential to assess the impact of intervention programs aimed at enhancing individuals’ knowledge and identification of food- and nutrition-related topics. To address this, the original articles developed by the Scopus search were carefully selected, focusing on those that specifically aimed to measure food or nutrition literacy before and after the implementation of an intervention program. To ensure the validity and reliability of the studies, only articles that used previously validated and consolidated tools for measuring food or nutrition literacy were considered. This analysis seeks to determine whether a disparity exists between theoretical discussions and practical measures employed to increase food or nutrition literacy levels. This study aimed to carry out a bibliometric analysis in the field of food and nutrition literacy to assess the current landscape of publications. Specifically, the goal was to define the profile of existing literature and determine whether the focus remains predominantly on theoretical concepts and definitions, or whether publications have progressed to reporting intervention outcomes aimed at improving levels of food and nutrition literacy. By examining the existing literature, this study seeks to identify any gaps or advancements in this area, thereby providing valuable insights for further research and evidence-based practices.

## 2. Methods

### 2.1. Data Source and Search Strategy

**Figure 1:**
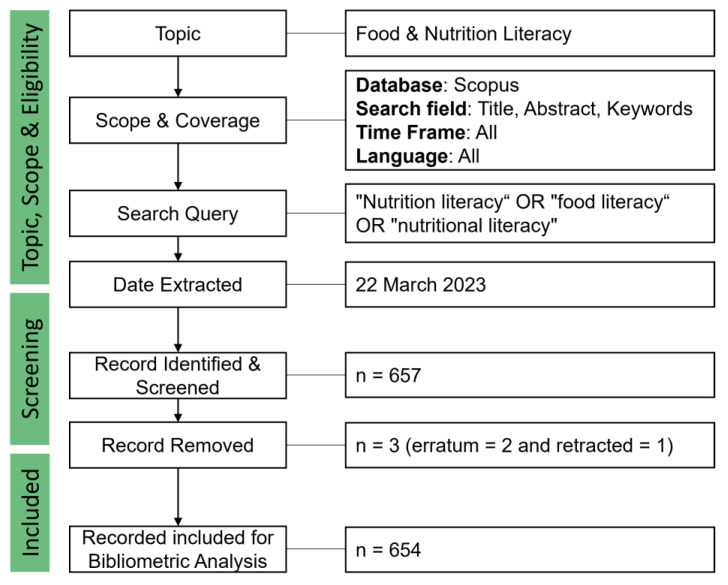
Flow diagram of the search strategy for bibliometric analysis. The query used in the title, abstract, and keywords was: “Nutrition literacy” OR “food literacy” OR “nutritional literacy”.

Bibliometric analysis was performed using the Scopus database as of March 2023. The query used was: (TITLE-ABS-KEY (“Nutrition literacy”) OR TITLE-ABS-KEY (“food literacy”) OR TITLE-ABS-KEY (“nutritional literacy”)) to search for relevant articles published in any language. Two erratum and one retracted document types were excluded to avoid double or false counting of documents (Figure 1).

### 2.2. Information Extraction

To prevent double counting and retracted papers that can lead to false-positive outcomes, errata materials were omitted from the analysis. A bibliometric analysis was performed for each document. The following tools were used: (i) VOSviewer (version 1.6.19) to create and visualize the bibliometric networks; (ii) Microsoft Excel to compute the frequencies and percentages of the published materials; and (iii) Harzing’s Publish and Perish program to compute the citation metrics. The Endnote 20 citation manager was used to import each citation.

### 2.3. Search Strategy and Study Selection

**Figure 2:**
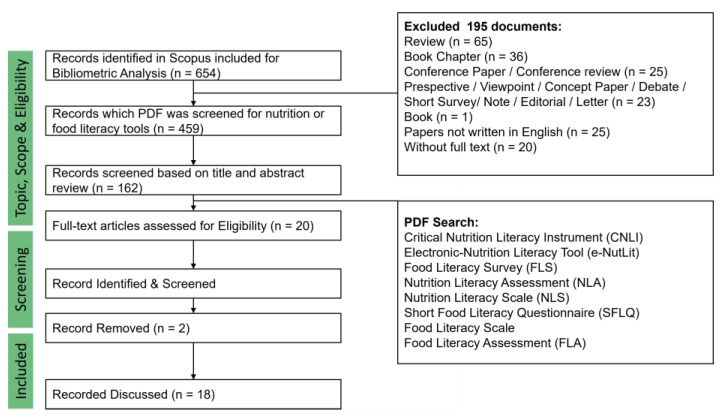
Original papers selection.

The primary source of literature was the one carried out in Scopus and described in the Section 2.1. A total of 459 English language papers reporting original data on food and nutrition literacy were identified by screening through an Endnote search, which involved searching the PDFs of articles for at least one of the following terms: Critical Nutrition Literacy Instrument (CNLI), Electronic-Nutrition Literacy Tool (e-NutLit), Food Literacy Survey (FLS), Nutrition Literacy Assessment (NLA), Nutrition Literacy Scale (NLS), Short Food Literacy Questionnaire (SFLQ), Food Literacy Scale, or Food Literacy Assessment (FLA). The study designs that sought to enhance a skill domain, such as functional, interactive, and critical, without or in conjunction with a cognitive domain (food/nutrition knowledge, attitude, and food/nutrition information understanding), and where the outcome was assessed using one of the instruments, were considered eligible (Figure 2).

Two reviewers (Rita Silva and Paula Silva) independently selected eligible papers based on their abstracts. The abstracts of all articles were reviewed, and articles that evaluated an intervention program aimed at increasing food or nutrition literacy and whose effectiveness was measured using at least one of the aforementioned tools were selected. The entire texts of all publications that could have been pertinent were then obtained and evaluated.

## 3. Results and Discussion

### 3.1. Description of Retrieved Literature

In total, 654 documents were retrieved from the Scopus database. Document types included journal articles, review articles, book chapters, conference papers, notes, editorials, letters, conference reviews, short surveys, and books. Most of the documents consisted of original articles, accounting for more than three-quarters of the total publications (502 documents, 76.77%). Review articles accounted for 65 documents (9.94%), while book chapters contributed to 36 documents (5.50%). The remaining document types comprised less than 5% of the total publications. The retrieved documents received a cumulative total of 7249 citations, indicating their impact and influence on the academic community. Furthermore, the h-index of the retrieved documents was 41, providing a measure of the significance and productivity of the research output.

Most retrieved documents were published in English, accounting for 96.18% of the total. Chinese was the second most prevalent language, representing 2.45% of documents. Additionally, a variety of other languages were used, including German, Spanish, Portuguese, Czech, Italian, Korean, Persian, and Turkish. Four documents were published in dual languages, indicating accessibility to a broader audience.

**Figure 3:**
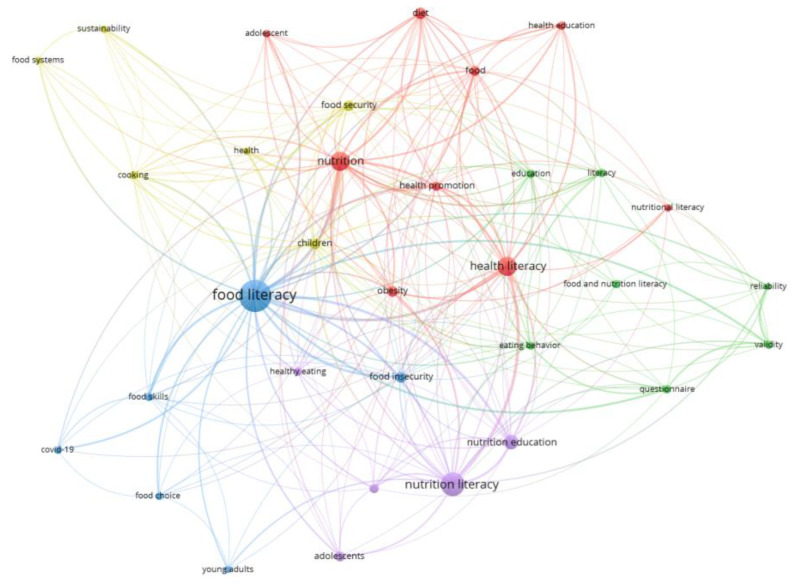
Author’s keyword network visualization. Unit of analysis = author keywords; counting method: full counting; minimum number of occurrences of a keyword = 10; cluster size = 5. The size of the circle of a keyword is determined by the number of occurrences. The query used in the title, abstract, and keywords was: “Nutrition literacy” OR “food literacy” OR “nutritional literacy”.

Mapping with the VOSviewer technique of author keywords with minimum occurrences of 10 showed that ones such as health literacy, nutrition, nutrition education, diet, children, food security, food insecurity, obesity, and food were the most encountered author keywords after the exclusion of “nutrition literacy” and “food literacy”, which are the keywords used in the search query (Figure 3). Circles of the same color signify that the papers have a common subject. Specifically, the red cluster (Cluster 1, 9 items) included keywords related to the influence of health literacy on the health-promoting behavior of adolescents with and without obesity. In the green cluster (cluster 2, seven items), the keywords point out studies related to the validation and use of nutrition literacy assessment instruments to measure nutrition literacy. Clusters 3 and 4 and the blue and yellow clusters included six keywords. Cluster 3 reports on the field of food insecurity, food skills, health literacy, and food preparation activities among young adults. Cluster 4 was related more to sustainable food systems and their reorientation towards healthy diets for children. Cluster 5, represented by purple, focuses on exploring the connection between nutrition knowledge, education, and various factors influencing adolescents’ dietary choices and lifestyle habits. Based on this ranking, researchers initially focused on comprehending the influence of health literacy on adolescents’ adoption of health-promoting behaviors. This knowledge can then be used to develop interventions aimed at promoting healthy eating and physical activity, considering the increasing concern surrounding the widespread occurrence of obesity and related chronic diseases among adolescents globally [11]. As nutrition education and intervention programs have been implemented, researchers have attempted to develop tools to accurately and reliably measure nutrition literacy, which is crucial for evaluating the effectiveness of those programs [12]. Later, scientific evidence recognized the complex interplay among food insecurity, food skills, health literacy, and sustainable food systems in shaping food choices and health outcomes among young adults. Some papers were then published to understand the broader socioeconomic and environmental determinants of food or nutrition literacy [13]. Scientific research on the factors that influence adolescents’ food intake and lifestyle habits may be considered relatively less studied than other topics.

### 3.2. Growth of Publications

**Figure 4:**
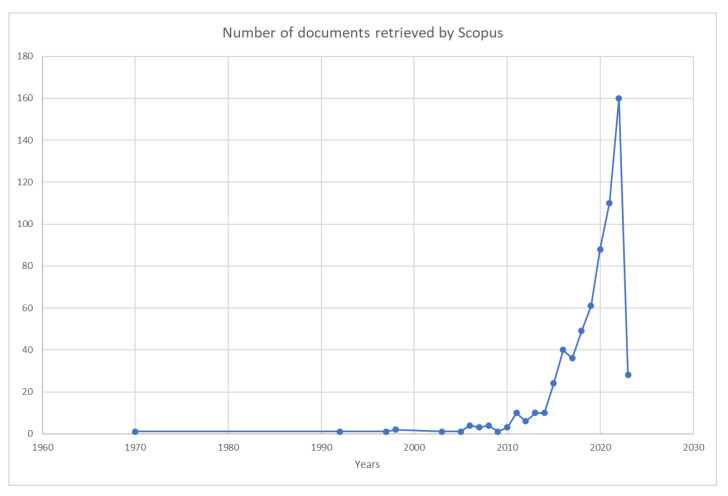
Growth of publications. The query used in the title, abstract, and keywords was: “Nutrition literacy” OR “food literacy” OR “nutritional literacy”.

The researcher can track the development and evolution of the study subject over time by looking at documents according to the year of publication. The first paper retrieved by Scopus was published in 1970 in the *Journal of Nutrition Education* and has the title “Nutritional literacy of high school students” and the authors are Johanna Dwyer, Jacob Feldman, and Jean Mayer. The goal of this first cross-sectional study was to evaluate nutrition instruction and knowledge among high school students in a major city [14]. The second article retrieved by Scopus was published in 1992. Papers were published every year after 2005 (Figure 4). However, this does not necessarily mean that no scientific documents related to food literacy or nutrition were produced before 1970 or during the gap years. By examining the cited bibliography list of Scopus-retrieved papers and the list of papers that cite them, it is apparent that such documents exist. The reason for the absence of publications during those years could be the fact of researchers did not use the terms “nutrition literacy” and “food literacy”. Studies defining these concepts appeared only in the early 2000s, such as Blitstein et al.’s (2006) [6] paper on nutrition literacy and Kolasa et al.’s (2001) [1] paper on food literacy.

The citation matrix for the retrieved documents per year after 2006 is presented in Table 1. The year with the most papers produced was 2022, with a total of 160 papers produced. This bibliometric study revealed a noticeable upward trend in the number of publications, particularly after 2018. It can be speculated that this increase is related to non-communicable diseases as primary targets for global disease prevention by the WHO, as shown in the 2016 Global Burden of Disease Study [15]. Furthermore, an implementation plan was introduced during the 2017 World Health Assembly. This plan aimed to provide guidance to countries to implement the six recommendations outlined by the Commission on Ending Childhood Obesity (2016). The recommendations specifically target the obesogenic environment and critical periods throughout the life course, aiming to address the issue of childhood obesity [16]. The total number of publications published was highest in the last three years (2019–2022); however, due to the short time that had elapsed since publications, those do not correspond to the years with the highest average citation per cited publication. Looking at Table 1, we must consider that the number of citations per publication was the highest for documents published in 2014, since in 2009, only one document was published and cited. In summary, the topic of food and nutrition literacy has gained significant attention since 2016, driven by mounting concerns regarding health issues associated with diet, such as obesity, diabetes, and heart disease. As individuals become more conscious of the significance of healthy eating, there is a heightened demand for research in the field of food and nutrition literacy. This increased interest and demand can account for the observed upward trend in publications and citations on this subject.

### 3.3. Authorship Pattern, Collaboration, and Prolific Authors

**Figure 5:**
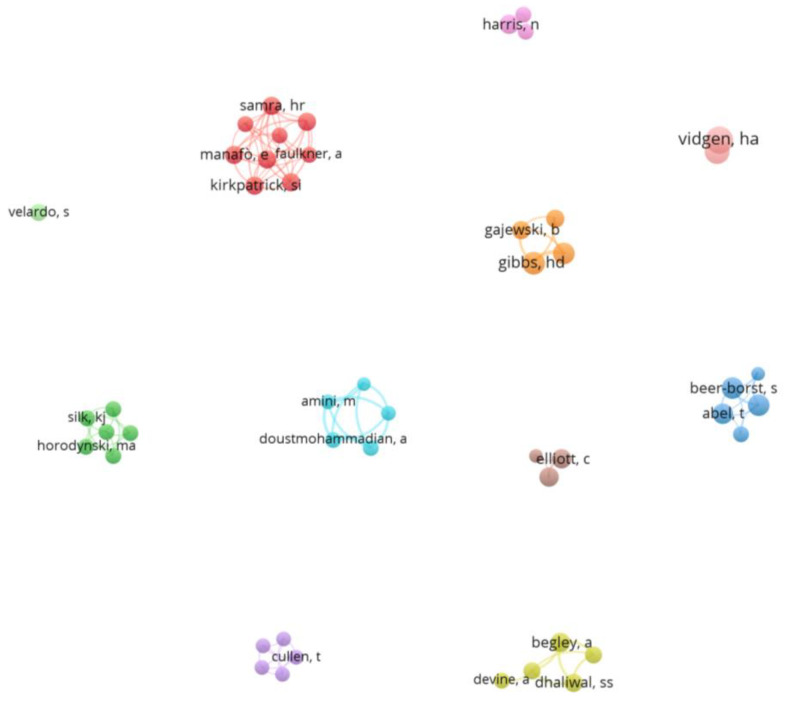
Network visualization map of co-authorship research. The size of the circle is determined by the total number of citations received by all documents published by the author. The distance between two authors in the visualization approximately indicates the relatedness of the authors in terms of co-authorship links. In general, the closer two authors are located to each other, the stronger their relatedness. The strongest co-authorship links between authors are also represented by lines. The query used in the title, abstract, and keywords was: “Nutrition literacy” OR “food literacy” OR “nutritional literacy”.

Based on the Scopus search results, 66 documents (10.15%) were single-author publications, while most of the documents (584; 89.85%) were collaborative efforts involving multiple authors. This indicates a high prevalence of team research or collaboration among researchers, with a collaboration rate of 89.85%. Figure 5 presents a network visualization map of co-authorship research, showing the relationships among the authors. The VOSviewer technique was employed to generate the map, focusing on authors who received a minimum of 50 citations. The resulting map consisted of 48 circles, with each circle representing an individual author. The stronger co-authorship relationship is among the Iranian authors (Azam Doustmohammadian, Nasrin Omidvar, Maryam Amini, Hassan Eini-Zinab, and Morteza Abdollahi). Followed by the co-authorship link between the two researchers at the University of Kansas Medical Center, Heather Gibbs, and Debra Kay Sullivan. As expected, stronger co-authorship links are among the authors of the same country.

Table 2 lists the top ten most productive authors. The most productive author with the highest number of publications was Satvinder Singh Dhaliwal (195 publications; 6695 citations), while Amanda Devine (169 publications; 5306 citations) and Debra Sullivan (133 publications; 4065 citations) ranked second and third, respectively. The most productive and cited researchers are not necessarily those with a higher recognition in the food and nutrition literacy research field. In fact, according to the Scopus search results the researchers more cited are Helen Anna Vidgen (6 documents, 177 citations), Danielle Gallegos (2 documents, 148 citations), and Heather Gibbs (14 documents, 127 citations), while Satvinder Singh Dhaliwal appears in 19th place (6 documents, 78 citations), Amanda Devine occupies the 33rd position (10 documents, 62 citations), and Debra Sullivan the 4th position (11 documents, 117 citations). The most cited paper is “Defining food literacy and its components” written by Helen Anna Vidgen and Danielle Gallegos, which helps explain why they are the most influential in the food and nutrition literacy research field. This paper has been highly cited because it provides a comprehensive and widely accepted definition of food literacy and provides significant contributions to the study of food literacy.

### 3.4. Geographical Distribution of Publications

**Figure 6:**
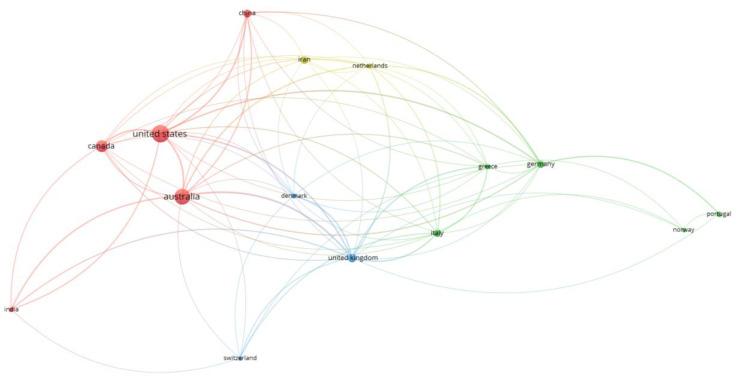
Network visualization map of international collaboration among countries with minimum productivity of 10 documents. The thickness of the connecting line between any two countries indicates the strength of collaboration. Countries with similar colors formed a single cluster. For example, countries with red color, such as the United States, Australia, Canada, China, and India, exist in one cluster. Germany, Greece, Italy, Norway, and Portugal are in green, while Denmark, Switzerland, the United Kingdom are in blue. Iran and the Netherlands are clustered in yellow. The query used in the title, abstract, and keywords was: “Nutrition literacy” OR “food literacy” OR “nutritional literacy”.

Researchers from 75 different countries contributed to the publication of the retrieved documents. The United States ranked first with a total of 150 (22.94%) documents, followed by Australia (18.96%) and Canada (10.70%). This agrees with topmost prolific authors results. The greater interest in food and nutrition literacy research in the United States and Australia may be related to the high rates of obesity and other diet-related health issues. In 2019, adult obesity rates in the United States reached 42.8%. In Australia, the adult obesity rate was 30.4% in 2017 [17]. Figure 6 displays a visualization describing collaboration between countries, specifically focusing on countries with a minimum productivity of 10 documents. The map shows a total of 15 countries that are divided into four distinct clusters, each represented by a different color. This visualization provides an overview of the collaborative relationships among these countries in the research endeavor.

### 3.5. Preferred Journals and Top Cited Documents

*Nutrients* ranked first with 52 documents, followed by the *International Journal of Environmental Research and Public Health* at second place (39 documents), both edited by Multidisciplinary Digital Publishing Institute (MDPI). The other top five journals are *Journal of Nutrition Education and Behavior* (30 documents), *Public Health Nutrition* (23 documents), and *Appetite* (20 documents).

The article that received the highest citation, a total of 509 citations, is “Defining food literacy and its components” published in the journal *Appetite*. This study aims to provide a comprehensive definition of the scope of the meaning of the term food literacy. The authors defined food literacy as “Food literacy is the scaffolding that empowers individuals, households, communities, or nations to protect diet quality through change and strengthen dietary resilience over time. It is composed of a collection of inter-related knowledge, skills and behaviors required to plan, manage, select, prepare, and eat food to meet needs and determine intake”. They also identified 11 components of food literacy derived from Expert and Young People’s Studies [4]. This is one of the most used definitions of food literacy that justifies the number of citations. The second most cited article, “From nutrients to nurturance: A conceptual introduction to food well-being” was published in the *Journal of Public Policy and Marketing* in 2011 and received a total of 300 citations. The authors argue that the traditional approach to nutrition, which focuses solely on the nutrient content of food, is limited in its ability to capture the complex relationship between food and health. Instead, they proposed a new concept called “food well-being”, which emphasizes the psychological, physical, emotional, and social relationship dimensions of food at both the individual and societal levels [2]. This paper has been highly cited and has been influential in shifting the focus of nutrition research to more holistic approaches that consider the broader context of food consumption. In the third place is the paper “Identifying factors that promote consumer behaviors causing expired domestic food waste” published in 2014 in the *Journal of Consumer Behaviour* with a total of 200 citations. Food waste is a major global issue, and this study provides valuable insights into the better understand the causes of consumer behaviors that result in expired domestic food waste. It identifies three specific core causal factors associated with higher levels of food waste: (i) food supply knowledge, (ii) food location knowledge, and (iii) food literacy. Overall, this study provides valuable insights into the complex nature of household food waste, and its practical recommendations make it a valuable resource that can be applied to interventions targeting behavioral change to reduce domestic food waste [18]. Hence, it is likely to be cited frequently in the food or nutrition literacy field.

### 3.6. Do Research Projects Promote Food/Nutrition Literacy?

From bibliometric research, it is possible to conclude that by conducting research, scientists and health professionals are trying to understand the relationship between diet and health, identify best practices for promoting healthy eating behaviors, and develop effective interventions for improving food/nutrition literacy. These interventions may include educational programs, policy changes, or other interventions designed to promote healthy eating habits. The aims of this section are to identify: (1) strategies and principal components of food and nutrition promotion, (2) the implementation methods of the interventions, and (3) the effectiveness of interventions in promoting food/nutrition literacy. A search of Endnote resulted in 162 original articles that mentioned at least one of the instruments measuring food or nutrition literacy. Of these, only 18 articles reported studies in which these instruments were used to measure the effects of intervention programs (Table 3). The following is a summary of these studies and their key findings.

Childhood is a critical period for the development of lifelong eating habits. Interventions targeting food literacy and nutrition can provide children with the knowledge and skills necessary for making healthy food choices. By investing in good eating habits early, these interventions can contribute to the prevention of diet-related diseases and promote overall health and well-being. “FOODcamp” is the name of a school-based intervention on food literacy, health literacy, and school well-being carried out among schoolchildren. The intervention included various food-related classes and activities. This study demonstrated that even a relatively brief intervention can have a positive impact on food literacy, indicating the program’s effectiveness in enhancing children’s knowledge and skills related to food. The results showed small but significant improvements in food literacy, particularly in the dimensions of “to do”, “to sense”, and “to know”. These dimensions refer to the practical skills, knowledge, and sensory experiences related to food. This suggests that future interventions should explore strategies that specifically target these food literacy dimensions. To gain comprehensive understanding, future studies should investigate whether the positive effects observed in the short term are sustained over time. It is crucial to explore whether the observed effect sizes in food literacy have meaningful implications for children’s dietary choices and overall food-related behaviors. This indicates the importance of understanding the practical significance and real-world impact of improving food literacy to promote healthier eating habits and lifestyle choices [19].

A cluster randomized controlled trial evaluated the impact of the PhunkyFoods intervention on the food literacy, cooking skills, and dietary behaviors of primary school children in Yorkshire, UK. The intervention included nutritional education activities delivered through school assemblies, classroom activities, and after-school clubs. The results showed that the intervention group had higher food literacy and cooking skill scores than the control group after 12 months. These results highlight the importance of interventions focused on nutrition and food literacy to enhance children’s understanding of essential nutrients, food groups, and benefits of consuming a diverse range of foods. Owing to the complex nature of the PhunkyFoods program and contextual factors, it is not feasible to evaluate its efficacy through experimental settings. Instead, the research perspective focuses on the effectiveness of theory-based approaches. A cluster randomized controlled trial design is employed, allowing for an investigation into what works and in what circumstances. However, the flexibility of schools to choose from the active ingredients in the Logic Model adds complexity and limits the statistical power for comparing outcomes between schools [20].

Food or nutrition literacy interventions can play a crucial role in reducing health disparities, particularly among disadvantaged populations. Children from low-income or marginalized backgrounds may face additional challenges in accessing nutritious food and understanding healthy eating practices. It is possible to address these disparities and improve health outcomes by implementing interventions specifically designed for these populations. A study conducted on Iranian Kurdish primary school children aimed to enhance food literacy through an intervention based on the intervention-mapping approach. The results demonstrated a significant difference in all aspects of food literacy between the control and intervention groups following the intervention. This study emphasizes the significance of school-based interventions and offers valuable insights into enhancing food literacy among children, especially in disadvantaged regions [21].

The intervention-mapping model, which uses both quantitative and qualitative methods, allows for a comprehensive understanding of the interconnections and complexities of social norms, human behavior, and social determinants of health. Incorporating multiple research approaches may provide a more nuanced and holistic perspective [22]. In conclusion, interventions for children’s food literacy have a positive impact, and it is important to engage children in practical food-related experiences.

Some interventions, rather than improving nutritional behaviors, aim to promote healthy lifestyles. One study explored how maternal eating habits and physical activity levels influence various aspects of food-related behaviors, physical activity practices, and gross motor development in preschoolers. The findings highlight the associations between mothers and children in terms of food habits, fruit and vegetable consumption, and other dietary items. This study emphasizes the importance of effective interventions targeted at both mothers and preschoolers in disadvantaged areas to promote healthy lifestyles [23]. The role of behavioral components and factors influencing behavior in personalized dietary interventions is very important. Assessing the behavior and factors influencing it during nutrition assessment can improve the effectiveness and outcomes of personalized dietary interventions. Behavioral components in nutrition assessment can enhance communication strategies, tailor interventions, and contribute to positive health outcomes [24].

Cooking programs are important to increase confidence in cooking, mental health, and other outcomes. Despite the difficulty in defining and measuring cooking confidence, the potential biases in self-report measures and sampling, and limitations in the study design, Rees J. et al. (2022) [25] found significant improvements in perceived general health, mental health, and subjective vitality among participants after a 7-week food literacy cooking program. Cooking confidence increased for all participants, with greater gains observed for males. Positive outcomes were maintained for six months after the program. However, there was no intervention effect on fruit and vegetable consumption, nutritional knowledge, or healthy eating. The study concludes that food literacy cooking interventions can have mental health benefits and suggests further research into their potential as preventive measures for population mental health programs. It recommends targeting population groups that will experience the greatest gains, addressing barriers to healthy eating, incorporating sustainability frameworks, providing education on the value of fresh whole foods, and linking research to governmental directives for greater impact on the community [25].

Azevedo et al. (2019) [26] tried a different intervention approach to increase the nutrition literacy of families. The intervention consisted of a web-based social network where participants interacted, accessed educational materials, used apps, and engaged in nutritional challenges focused on fruits, vegetables, sugar, and salt. This study was conducted in 37 kindergartens in Portugal, involving 877 families. The main outcome measure was parental nutrition literacy, which was assessed through a self-reported survey with four dimensions: nutrients, food portions, Portuguese food wheel groups, and food labeling. The analysis utilized a general linear model with repeated measures to examine the effect of the intervention on nutrition literacy scores. The results showed that families actively participated in the intervention by uploading recipes, photographs of challenges, and other items on the interactive platform. The intervention group (*n* = 106) demonstrated a significantly higher mean score of nutrition literacy at the end of the program than at baseline, regardless of parental education and perceived income status. By contrast, no significant differences were observed in the scores of the control group (*n* = 83). The study suggests that the web-based gamification program, known as the Nutriscience Project, provides families and classroom staff with significant information on best practices for intervention in families and schools. The digital and entertaining interactive platform appeared to be a useful and easily adaptable educational tool for promoting healthy eating. The authors recommend that future implementations of the Nutriscience Project should consider longer intervention periods, ideally lasting for a minimum of five months. They also suggested evaluating the dietary habits of both families and school communities before and after the intervention to assess the impact of the intervention. Additionally, they proposed exploring interaction markers with the platform, such as exposure time, to gain further insight. Furthermore, the authors emphasized the need for additional research to determine the effectiveness of gamification in the realm of health and well-being. This entails conducting well-designed studies that compare gamified and non-gamified interventions, exploring various gamification approaches with and without ecological designs, and conducting long-term follow-up assessments of outcomes. Moreover, future research should investigate social interaction dynamics within families, between families, and between families and schools, and examine how these interactions influence participants’ nutrition literacy [26].

“Nutricity” shows promise as a bilingual mobile nutrition literacy intervention for parents of young children, which can be feasibly introduced in pediatric clinics. The findings from the study carried out by Gibbs et al. (2018) indicate strong support and likability of “Nutricity” among parents across various levels of nutrition literacy and language groups. Pediatric clinic personnel also confirmed the feasibility of incorporating “Nutricity” as a wait-time intervention within clinics, recognizing its potential to deliver preventive education, which is often lacking in routine patient interactions. The nutrition literacy deficits of the parent sample, as indicated by NLit scores, highlight the successful recruitment of the target population. While differences in nutrition literacy were observed between language groups, national assessment data suggest that low health literacy is more common among Hispanics and is potentially influenced by socioeconomic and educational factors. Clinical provider feedback emphasized Nutricity’s suitability as a vehicle for delivering educational material during waiting times, leading to improved patient satisfaction and increased opportunities for patient-provider communication. With the increasing popularity of health promotion apps and the widespread use of smartphones among the population, Nutricity’s design for any Internet-accessing device allows for continuous learning beyond the clinic setting. By introducing “Nutricity” as a wait-time intervention, it has the potential to overcome the challenge of engaging parents in nutrition interventions, as they are already active participants in their child’s healthcare. However, further research is needed to explore the effectiveness of using developmentally appropriate games to engage young children and to assess their impact on knowledge and behavior improvement [27].

Social media offers a powerful platform to promote food/nutrition literacy by providing widespread access to educational resources, fostering community engagement, influencing consumer behaviors, and facilitating behavior change. It has the potential to empower individuals to make healthier food choices, improve cooking skills, and enhance their overall nutritional knowledge. In a cross-sectional survey conducted in Flanders, Belgium, involving 1002 adolescents aged 11–19 years from 18 secondary schools, the study findings indicated a positive association between self-reported exposure to food marketing and overall food messages on social media and eating attitudes, behaviors, perceived norms, and food literacy among adolescents. However, the relationship between food exposure and intake varies depending on the type of food. Specifically, this study found that exposure to non-core food messages on social media had a positive relationship with non-core food intake, and this association was mediated by descriptive norms. In contrast, exposure to core food messages on social media had a positive relationship with core food intake, and this association was mediated by food literacy. These results highlight the impact of food marketing and social media messages on adolescents’ eating patterns and behaviors, as well as the role of norms and food literacy in mediating these associations. This study highlights the significance of social media in adolescents’ eating behaviors. These findings suggest that health professionals have an opportunity to use social media as a platform to promote healthy eating, particularly core foods, among adolescents. The study also calls for relevant policy actions to regulate the marketing of non-core foods to adolescents on social media [28].

Social media interventions to increase food literacy should not be limited to adolescents but should also be directed towards adults. By utilizing social media platforms, these interventions can effectively educate and empower adults to make healthier food choices, develop essential skills, and improve their overall nutritional wellbeing. Ng et al. (2022) [29] evaluated the effectiveness of a 4-week online intervention delivered through social media on food literacy and fruit and vegetable consumption in Australian adults. The intervention called the “online MedDiet challenge”, was delivered via Facebook and included the sharing of infographics, recipes, and informational videos aligned with food literacy concepts related to the Mediterranean Diet. This study used a pre–post single-group experimental design with 29 participants. The outcome measures included a validated food literacy questionnaire and a recording of fruit and vegetable consumption by using questions from the National Nutrition Survey. The average age of participants in this study was 52 years. The findings revealed significant improvements in post-intervention food literacy, with enhancements ranging from 21% to 45% across the various survey components. Additionally, participants reported an increase in daily fruit and vegetable consumption, with an average rise of 0.6 servings for fruits and 1.3 servings for vegetables. These results suggest that social media platforms have the potential to positively impact adult fruit and vegetable consumption by enhancing food literacy skills [29]. In summary, regardless of age, social media have the potential to influence eating outcomes and promote food literacy.

Intervention programs aimed at promoting healthier food-shopping behaviors have proven to be effective in improving consumer choices and overall dietary quality. These programs can utilize various strategies such as providing nutrition education, implementing front-of-package labeling systems, and leveraging digital technologies to facilitate informed decision-making. The use of digital front-of-package labels (FoPL) to support healthy food choices in online supermarkets has not been widely studied, despite their advantages in scalability, personalization, and ease of implementation. Fuchs et al. (2022) [30] developed a web browser extension that displayed the Nutri-Score FoPL in a real online supermarket. In a randomized controlled trial with 135 participants, those exposed to the digital FoPL intervention made grocery choices with higher nutritional quality, including lower sugar and saturated fat contents. Participants with low food literacy benefited from the use of digital labels. Moreover, the participants expressed strong support for the introduction of Nutri-Score labels. The findings suggest that implementing digital FoPLs in online supermarkets can positively influence consumers’ food choices regardless of their level of food literacy and enjoy widespread approval. This study provides further evidence for the implementation of interpretive FoPLs, such as Nutri-Scores [30]. A study aimed to compare the effectiveness of computer-based virtual reality (CBVR) and voice-over PowerPoint (PP) presentations on nutrition literacy (NL) and healthy food purchasing self-efficacy (HFPSE), showing that both groups experienced significant improvements in HFPSE, which were sustained at the three-month follow-up. These findings suggest that CBVR is as effective as voice-over PP in enhancing HFPSE. This study highlights the potential of CBVR technology in future nutrition education programs as it can achieve learning outcomes similar those to of traditional presentations. The limitations of this study include the small sample size, lack of population diversity, and shortened NL assessment tool. Nonetheless, the findings of this study indicate that CBVR can improve HFPSE in selecting healthy food options and maintain these improvements over time. The accessibility and affordability of CBVR make it a promising tool for nutrition education, enabling the dissemination of credible nutrition information to a wider range of consumers [31].

The Prescription for Health farmers’ market fruit and vegetable (FV) prescription program, implemented in a rural, low-access, low-income community in Michigan, aims to address food insecurity and improve nutrition by connecting healthcare and food systems. The program enrolled 33 adult participants with chronic diseases and provided them with weekly farmers’ market FV vouchers, educational nutrition handouts, and seasonally healthy recipes over 10 weeks. While most measured metrics showed minimal change, one notable and significant finding was the improvement in the participants’ quality of life. Informal participant feedback highlighted increased social interaction as a result of attending the farmers’ market, which led researchers to recognize the potential of farmers’ markets to foster meaningful social connections among participants and farmers. Considering the prevalent issues of loneliness and social isolation, this finding suggests that farmers’ market prescription programs can serve as an innovative form of nature-based social prescribing. This study discusses the health outcomes of the Prescription for Health pilot program, reflects on the unique aspects of implementing it in a rural area, and explores future opportunities for similar initiatives [32].

A longitudinal intervention study focusing on the role of food literacy (FL) in improving dietary behavior among German office workers implemented a comprehensive 3-week workplace health promotion program (WHPP) that aimed to provide participants with in-depth knowledge and skills regarding nutrition and health. The results indicated significant and strong improvements in both FL and dietary intake (DI) following WHPP compared with baseline. These improvements were observed in both the short- and long-term, with FL showing a significantly moderate effect on DI across all measurement time points. These findings suggest that well-designed WHPPs can lead to long-term enhancements in FL and DI, highlighting the importance of FL in expanding food-related knowledge and skills to promote healthier dietary behaviors. This study provides valuable long-term insights into the role of FL in WHPPs, and supports the incorporation of FL in the development of comprehensive workplace health programs to improve dietary intake [33].

Nutritional interventions tailored to address nutrition literacy deficits can have a positive impact on improving dietary behaviors in outpatient nutrition clinics. An intervention arm study, in which dietitians received patient nutrition literacy levels and tailored interventions, accordingly, showed greater improvements in dietary behaviors than the control arm. Specifically, patients in the intervention group demonstrated improvements in 10 out of 25 measured diet behaviors, with increased consumption of green vegetables also reported. These findings indicate that considering nutrition literacy during outpatient nutrition consultations can inform the dietitian’s intervention approach and lead to better outcomes in terms of dietary behavior. The implications of this pilot study for research and practice are significant. This highlights the importance of incorporating nutrition literacy assessments into nutrition consultations to guide interventions and enhance the effectiveness of patient-centered nutrition care [34].

Intervention programs designed to increase food/nutrition literacy among cancer patients and survivors play a crucial role in supporting their overall well-being and improving treatment outcomes. Both food and nutrition literacy interventions provide individuals with the knowledge and skills necessary to make informed dietary choices, manage nutritional challenges during and after cancer treatment, and to adopt healthy eating habits. A feasibility study on nutrition education workshops for breast cancer survivors, known as the Healthy Eating and Living Against Breast Cancer (HEAL-BCa) study, aimed to address insufficient knowledge of nutrition among breast cancer patients. The study enrolled 59 female breast cancer patients who had completed treatment and randomized them into intervention and control groups. The intervention group participated in six nutrition education sessions, while the control group received brochures as an intervention. Measurements were conducted at two time points: baseline and the 3-month follow-up. The measurements included assessments using the Assessment Instrument for Breast Cancer (NLit-BCa), screeners for fruit/vegetable and general health literacy, and height and weight measurements. The study showed high follow-up rates, and although the effect sizes on nutrition literacy varied, the intervention was acceptable. The findings support the feasibility of scaling up this intervention to benefit breast cancer survivors. Future steps include testing it in more diverse populations and incorporating participant feedback to improve content [35]. A pilot study examined the feasibility of a weight-management program for overweight men with localized prostate cancer. The comprehensive program resulted in significant weight loss, improvements in diet quality, and favorable cardiometabolic effects. The findings suggest that a coaching intervention can lead to substantial weight loss and positive health outcomes in overweight men preparing for prostatectomy [36]. Overall, these studies underscore the importance of addressing nutrition literacy and providing targeted interventions for cancer populations. Enhancing nutritional knowledge and promoting healthy eating behaviors among cancer patients and survivors can contribute to optimizing treatment outcomes, improving quality of life, and reducing the risk of cancer recurrence and associated complications.

In conclusion, the studies reviewed in this bibliometric study provide evidence that interventions targeting food/nutrition literacy have a positive impact on individuals, particularly children and families. These interventions aim to improve knowledge and skills related to food and nutrition, promote healthy eating behaviors, and enhance overall well-being. These findings suggest that school-based interventions, web-based platforms, social media, and digital tools can effectively enhance food/nutrition literacy and improve dietary choices. Overall, these findings highlight the importance of investing in food/nutrition literacy interventions to promote healthy eating habits, prevent diet-related diseases, and reduce health disparities. Further interventions are needed to increase food/nutrition literacy, which should be well-structured based on the extensive existing literature. These interventions should be assessed using validated instruments that have shown robustness and accuracy in their results. It is time to move from theory to action.

## 4. Conclusions

In recent years, there has been a remarkable surge in research focusing on food and nutrition literacy, indicating a growing recognition of its importance. Despite this recognition, limited research has been conducted on food and nutrition literacy intervention programs. Without effective intervention programs, the practical application of food and nutrition literacy remains limited, and its impact on improving eating habits and public health outcomes is questionable. It is crucial to address this gap and to conduct further research to identify effective interventions and approaches that contribute to different populations and settings. An analysis of the most cited articles revealed the profound impact of studies that provide comprehensive definitions of food and nutrition literacy and introduce holistic approaches. However, we should be concerned about the generalizability of existing intervention programs. If the effectiveness of these programs is not sufficiently tested across different contexts, it is uncertain whether they can be successfully implemented on a larger scale. These studies emphasize the broader dimensions of food and their intricate relationships with overall well-being. They have played a pivotal role in reshaping the understanding of food/nutrition literacy and redirecting research towards more comprehensive and practical perspectives. To bring about meaningful changes, there is an urgent need for additional research to improve policies and practices in this critical public health area. It is imperative to bridge the gaps in defining and measuring food and nutrition literacy and identify interventions that are truly effective. Moreover, it is essential to consider the broader context and societal implications of food consumption. Improving individual knowledge and skills may not be sufficient to overcome the barriers created by factors such as food accessibility, affordability, and marketing tactics employed by the food industry. Therefore, a critical perspective should consider the broader context and societal implications of food consumption, including the need for policy change and structural intervention. In doing so, we can promote healthy eating habits and effectively address diet-related health issues. To ensure the success of food/nutrition literacy programs, it is crucial to measure their outcomes and impact. By evaluating these results, we can assess the effectiveness of these programs and make informed decisions to enhance their success. This emphasis on measurement underscores the importance of evidence-based approaches and the need for continual improvements in food and nutrition literacy initiatives. Moreover, it is necessary to discuss the challenges and limitations of conducting research to measure intervention program efficacy and enhance policies and practices. Evaluating the impact of intervention programs can be complex, requiring rigorous study designs and long-term follow-up.

In conclusion, this study highlights the critical need for practical action to enhance food and nutrition literacy. It emphasizes the importance of not only conducting research and identifying effective interventions but also bridging the gap between research and practice. These findings underscore the significance of implementing programs, changing policies, and measuring outcomes to make meaningful progress in promoting healthy eating habits and addressing diet-related health issues. By focusing on the practical aspects of food and nutrition literacy, we can translate knowledge into action that has a tangible impact on public health. Through these practical efforts, policies, practices, and interventions can be improved, ultimately leading to positive changes in individuals’ dietary behaviors and overall well-being.

## Figures and Tables

**Table 1:** Annual number of publications and citation matrix.

Year	TP	NPC	TC	C/P	C/CP	h
2006	4	4	128	32.00	32.00	4
2007	3	2	76	25.33	38.00	2
2008	4	4	158	39.50	39.50	3
2009	1	1	86	86.00	86.00	1
2010	3	3	56	18.67	18.67	3
2011	10	9	545	54.50	60.56	7
2012	6	6	99	16.50	16.50	5
2013	10	10	304	30.40	30.40	9
2014	10	9	755	75.50	83.89	8
2015	24	24	818	34.08	34.08	14
2016	41	37	571	13.93	15.43	11
2017	36	32	772	21.44	24.13	14
2018	49	47	743	15.16	15.81	16
2019	61	56	718	11.77	12.82	15
2020	88	74	631	7.17	8.53	14
2021	110	88	459	4.17	5.22	10
2022	160	85	195	1.22	2.29	6
2023	27	4	5	0.19	1.25	1

Notes: The query used in the title, abstract, and keywords was: “Nutrition literacy” OR “food literacy” OR “nutritional literacy”. TP: total number of publications; NCP: number of cited publications; TC: total citations; C/P: average citations per publication; C/CP: average citations per cited publication; and h: h-index.

**Table 2:** Top 10 most productive authors.

Name	Affiliation	Country	TP	TC	C/P	h
Satvinder Singh Dhaliwal	The Faculty of Health Sciences	Australia	195	6695	34.33	43
Amanda Devine	Edith Cowan University	Australia	169	5306	31.40	43
Debra Kay Sullivan	University of Kansas Medical Center	United States	133	4065	30.56	34
Nasrin Omidvar	National Nutrition and Food Technology Research Institute, Shahid Beheshti University of Medical Sciences	Iran	103	1221	11.85	20
Hassan Eini-Zinab	Shahid Beheshti University of Medical Sciences	Iran	85	827	9.73	18
Andrea M Begley	Curtin University	Australia	50	671	13.42	16
Azam Doustmohammadian	Iran University of Medical Sciences	Iran	44	244	5.55	8
Maryam Amini	National Nutrition and Food Technology Research Institute, Shahid Beheshti University of Medical Sciences	Iran	43	401	9.33	12
Heather D. Gibbs	University of Kansas Medical Center	United States	27	334	12.37	8
Lucy Meredith Butcher	Edith Cowan University	Australia	14	209	14.93	8

Notes: TP: total number of publications; TC: total citations; C/P: average citations per publication; and h: h-index. The query used in the title, abstract, and keywords was: “Nutrition literacy” OR “food literacy” OR “nutritional literacy”.

**Table 3:** Original papers aimed to measure the effects of an intervention program aimed at increasing food or nutrition literacy.

Name	Number	Selected	DOI
Papers retrieved by Scopus	654		
Original papers	459	18	
Original papers: Critical Nutrition Literacy Instrument	0	0	
Original papers: Electronic-Nutrition Literacy Tool	2	0	
Original papers: Nutrition Literacy Assessment	54	8	10.25122/jml-2019-0025;
			10.1016/j.jneb.2018.10.008;
			10.3389/fnut.2018.00129;
			10.1080/01635581.2020.1856890;
			10.1016/j.clnesp.2019.03.017;
			10.1016/j.jneb.2021.07.013;
			10.1007/s13187-017-1238-z;
			10.1177/08901171221108274
Original papers: Nutrition Literacy Scale	23	0	
Original papers: Short Food Literacy Questionnaire	10	2	10.3390/nu14102044;
			10.3390/ijerph192416534
Original papers: Food Literacy Scale	32	3	10.1111/hsc.13909;
			10.1017/S1368980021003116;
			10.1016/j.appet.2021.105845
Original papers: Food Literacy Assessment	23	4	10.1186/s13063-022-06558-5;
			10.3390/ijerph18062875;
			10.1177/15248399221093966;
			10.3389/fpubh.2022.1059677
Original papers: Food Literacy Survey	7	1	10.3389/fnut.2022.802940

## Data Availability

The data used to support the findings of this study can be made available by the corresponding author upon request.
